# Characterization of LIMA1 and its emerging roles and potential therapeutic prospects in cancers

**DOI:** 10.3389/fonc.2023.1115943

**Published:** 2023-05-19

**Authors:** Xiaoxiao Wang, Chao Zhang, Huangqin Song, Junlong Yuan, Xiaomin Zhang, Yiran Yuan, Lei Zhang, Jiefeng He

**Affiliations:** ^1^Third Hospital of Shanxi Medical University, Shanxi Bethune Hospital, Shanxi Academy of Medical Sciences, Tongji Shanxi Hospital, Taiyuan, China; ^2^Department of Hepatobiliary Surgery, Shanxi Bethune Hospital, Shanxi Academy of Medical Sciences, Tongji Shanxi Hospital, Third Hospital of Shanxi Medical University, Taiyuan, China; ^3^Hepatic Surgery Center, Institute of Hepato-Pancreato-Biliary Surgery, Tongji Hospital, Tongji Medical College, Huazhong University of Science and Technology, Wuhan, China

**Keywords:** actin cytoskeleton, LIMA1, cells migration and metastatic, EMT, angiogenesis, cancer progression

## Abstract

Actin is the most abundant and highly conserved cytoskeletal protein present in all eukaryotic cells. Remodeling of the actin cytoskeleton is controlled by a variety of actin-binding proteins that are extensively involved in biological processes such as cell motility and maintenance of cell shape. LIM domain and actin-binding protein 1 (LIMA1), as an important actin cytoskeletal regulator, was initially thought to be a tumor suppressor frequently downregulated in epithelial tumors. Importantly, the deficiency of LIMA1 may be responsible for dysregulated cytoskeletal dynamics, altered cell motility and disrupted cell-cell adhesion, which promote tumor proliferation, invasion and migration. As research progresses, the roles of LIMA1 extend from cytoskeletal dynamics and cell motility to cell division, gene regulation, apical extrusion, angiogenesis, cellular metabolism and lipid metabolism. However, the expression of LIMA1 in malignant tumors and its mechanism of action have not yet been elucidated, and many problems and challenges remain to be addressed. Therefore, this review systematically describes the structure and biological functions of LIMA1 and explores its expression and regulatory mechanism in malignant tumors, and further discusses its clinical value and therapeutic prospects.

## 1 Introduction

Actin-binding proteins mediate the assembly of actin monomers into distinct filamentous structures. Actin filaments assemble into a variety of networks and bundles that interconnect to form the actin cytoskeleton (1). This is a highly dynamic structure and the dynamic rearrangement of the actin cytoskeleton is fundamental to support a wide range of cellular behaviors. It plays a key role in cell biological processes including cell polarity, adhesion and migration, cell division, intracellular transport and endocytosis (2, 3). Recent studies showed that LIM structural domain proteins are associated with mediating cytoskeletal homeostasis and coordinating these cellular behaviors (4). LIM domain and actin-binding protein 1 (LIMA1) is an actin-binding cytoskeletal protein containing a LIM structural domain. It is subcellularly localized to actin stress fibers and focal adhesion plaques (5, 6). The amino-terminal and carboxy-terminal ends of LIMA1 are located lateral to the central LIM structural domain. They are present in at least two actin-binding domains that are capable of cross-linking and stabilizing cytoskeletal filaments and promoting stress fiber formation (7). This ensures cytoskeletal homeostasis and maintains integration and coordination between different actin regulatory pathways to support dynamic cellular behaviors.

LIMA1 is also known as epithelial protein lost in neoplasms (EPLIN) and sterol regulatory element binding protein 3 (SREBP3). It was initially identified as a differentially expressed gene in oral epithelial cell carcinogenesis using cDNA differential analysis (8). Subsequently, Maul et al. (9) first described and identified LIMA1 as a novel cytoskeletal protein. LIMA1 exists as two distinct isoforms: LIMA1-α containing 600 amino acids and LIMA1-β containing 759 amino acids (10). The amino acid structural domain sequence of LIMA1 is unique. The LIM domain-containing protein as an interaction site for specific signal transduction proteins can form two closely spaced zinc-binding subdomains and allow LIMA1 to dimerize on its own or bind to other proteins (5, 11). Due to the importance of LIMA1 in regulating actin cytoskeleton dynamics and its potential involvement in cadherin-mediated cell adhesion (12), the loss of LIMA1 from cancer cells may affect cell behavior and further enhance invasive characteristics. LIMA1 deficiency may be responsible for dysregulated cytoskeletal dynamics, altered cell motility and disrupted cell-cell adhesion, which can promote tumor proliferation, invasion, and migration (13).

Recent studies, including those from our laboratory, demonstrate a broader role for LIMA1 in controlling cancer cell behaviors. The effects of LIMA1 also extend from cell migration and cytoskeleton dynamics to cell cycle, gene regulation, angiogenesis, and lipid metabolism, among others, providing new ideas for future exploration of cancer treatment strategies (14). In this paper, the latest research progress on the molecular characteristics, biological functions and regulatory mechanisms of LIMA1 could be expounded. It focuses on the roles and significance of LIMA in various cancers and provides insights into its future research prospects.

## 2 The characterization and functions of *LIMA1*

### 2.1 The structure of *LIMA1*

**Figure 1:**
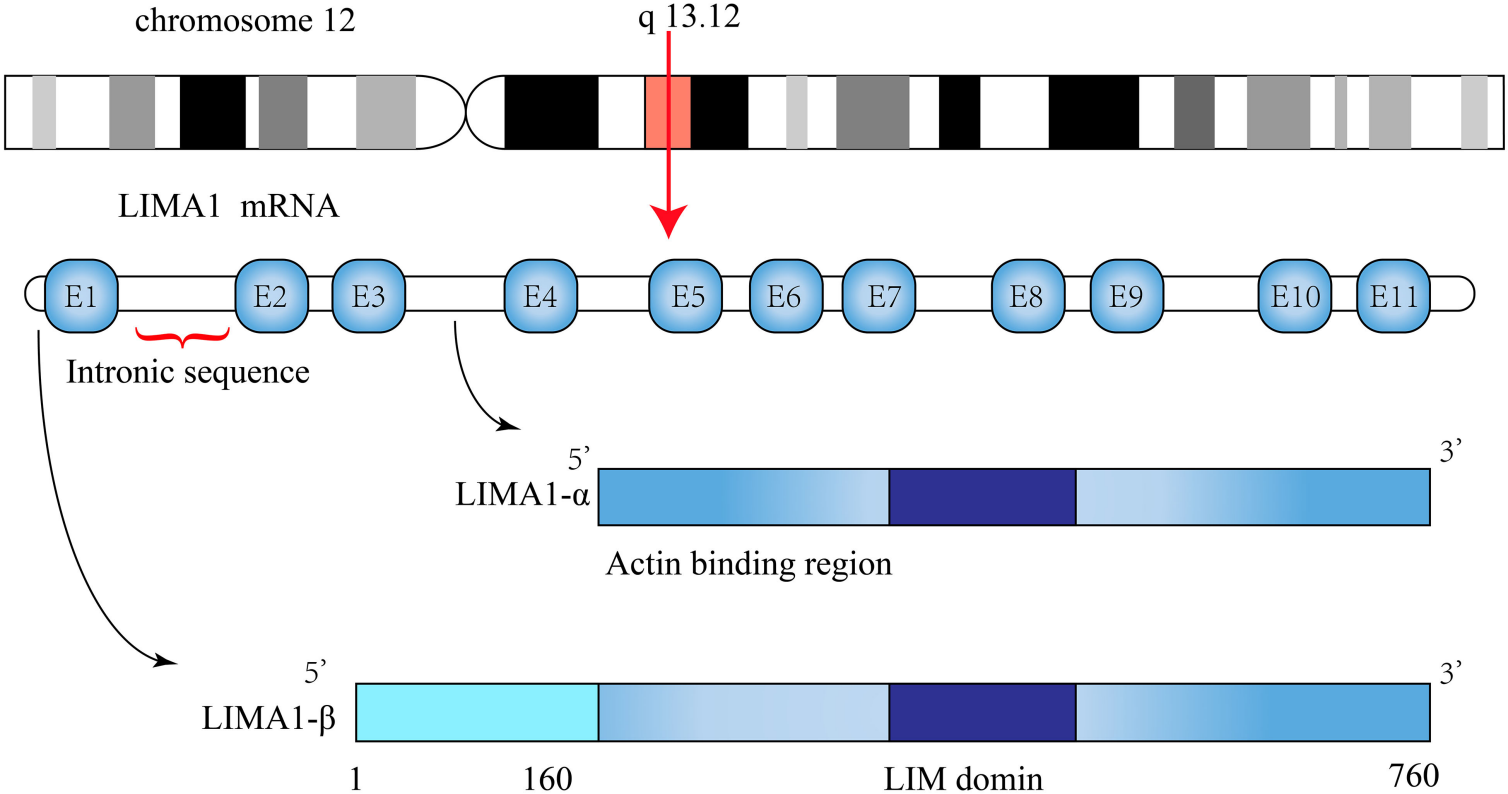
Schematic representation of *LIMA1* structure. The *LIMA1* gene is located on chromosome 12 and consists of 11 exons and 10 introns. Its protein contains two isoforms, a *LIMA1-α* of 600 amino acids and a *LIMA1-β* of 759 amino acids.

**Figure 2:**
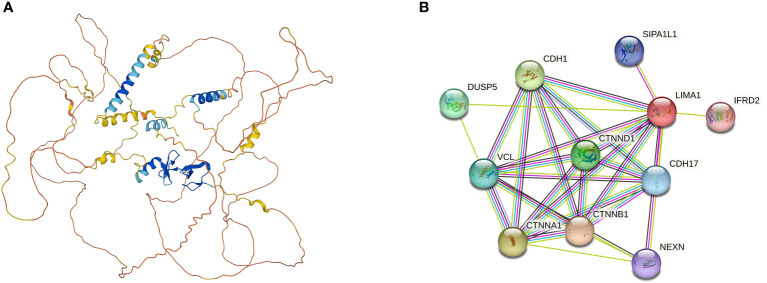
**(A)** Three-dimensional structure of *LIMA1* (PDB ID=2Y), from *AlphaFold Protein Structure Database* (https://alphafold.ebi.ac.uk/). *LIMA1* contains a LIM structural domain in the center and multiple actin-binding domains at the lateral end, which is capable of cross-linking and stabilizing a network of stabilized actin filaments. **(B)** Potential molecules and functional proteins interacting with *LIMA1*, which can be derived from the search tool *STRING* (http://www.string-db.org/) for retrieving interacting genes or proteins. DUSP5, Dual Specificity Phosphatase 5; CDH1, Cadherin 1; VCL, Vinculin; CTNND1, Catenin Delta 1; CTNNA1, Catenin Alpha 1; CTNNB1, Catenin Beta 1; SIPA1L1, Signal Induced Proliferation Associated 1 Like 1; CDH17, Cadherin 17; NEXN, Nexilin F-Actin Binding Protein; IFRD2, Interferon Related Developmental Regulator 2.

Current studies have identified the organization of the human LIMA1 gene, which is located on chromosome 12q13.12 and has a sequence length of 107,733 bp (5, 10). It consists of 11 exons spanning 100 kb and 10 introns. The LIMA1 gene is structured with two independent promoter regions. The DNA sequence at the transcription start site stimulates the expression of the promoter reporter gene constructs (13). Under the protection of 5*’* RACE and S1 nuclease, the transcription start site of LIMA1-α mRNA is approximately 50 kb downstream near the end of intron 3 and is positioned before exon 4 and contains 4-11 exons. Similarly, the transcription start site of LIMA1-β mRNA is located near the start of exon 1 of the gene and contains all 11 exons (10) (**Figure 1**). The amino acid sequences of these two isoforms are characterized by the presence of a centrally located LIM structural domain. The LIM structural domain is a cysteine-rich double zinc finger structural domain derived from the three homologous structural domain-containing transcription factors Lin-11, Isl-1 and Mec-3 (15, 16). The N-terminal and C-terminal sides of the LIM structural domain of LIMA1 can promote parallel formation of actin filament structures by cross-linking and binding actin filaments (7) (**Figure 2**).

Sequence analysis found that LIMA1-α and LIMA1-β isoforms are conserved across species (10). In subsequent working study, Maul et al. (17) isolated and characterized mouse and zebrafish LIMA1 homologous to humans. Akin to the human gene, both 593 aa LIMA1-α and 753 aa LIMA1-β isoforms were found in mouse, showing 77% and 75% identity with human isoforms (5, 17). In contrast, there was only one form in zebrafish, namely 629 aa LIMA1. The overall similarity between zebrafish and human amino acid sequences was not significant. Wang et al. (18) revealed two isoforms of the porcine LIMA1 gene with different expression patterns in muscle development and maintenance. LIMA1-α was preferentially expressed in developing skeletal muscles. Especially in the early stages, LIMA1-α played an important role in the process of muscle cell morphology and growth. LIMA1-β was not expressed during muscle development and only weak expression was detected in adult muscles (13, 18). Recent studies indicated that LIMA1-β regulated the distribution of OB-cadherin, which was involved in the assembly of cadherin-catenin protein complexes in osteoblasts, and affected bone formation (19).

### 2.2 The functions of LIMA1

#### 2.2.1 Controlling for actin dynamics

LIMA1 controls actin dynamics by stabilizing the actin filament network. The expression of LIMA1 increases the number and size of actin stress fibers and inhibits active Rac-induced membrane folding (6). Furthermore, LIMA1 inhibits actin filament nucleation and F-actin depolymerization by mediating the actin-related protein complex 2/3 (Arp2/3) (13). Moreover, Han et al. (7) showed that LIMA1 was a novel extracellular signal-regulated kinase (ERK)/mitogen-activated protein kinase (MAPK) substrate and that ERK responded, both *in vitro* and *in vivo*, to LIMA1 Ser360, Ser602, and Ser692 phosphorylation. ERK-mediated phosphorylation of LIMA1 decreased C-terminal region binding activity to actin leading to reorganization of actin filaments and enhanced cell motility. In addition, Song et al. (20) found that LIMA1 was bound to the actin cytoskeleton and inhibited anchorage-dependent growth of transformed NIH3T3 cells. LIMA1 exhibited a patchy pattern of distribution in the cytoplasm of Ras-transformed cells and activated Ras prevented or altered LIMA1 co-localization with actin stress fibers (21). These changes in subcellular localization of LIMA1 may represent a non-specific outcome of transformation, which frequently altered cell morphology and cytoskeleton. In this process, LIMA1-α is transcriptionally regulated by G-actin and megakaryoblastic acute leukemia (MAL)/myocardin related transcription factor (MRTF) coactivators. It is sensitive to drugs that can stabilize the inhibitory actin MAL complex and non-polymerizable actin mutants (22).

LIMA1 binds to α-catenin and mediates the interaction of the cadherin-catenin complex with F-actin, stabilizing the actin fibers and establishing the adhesion bands at the cell-cell junctions (12). Epithelial cells remodel their junctional structure by responding to mechanical forces. E-cadherin acts as a mechanosensitive regulator of this process of epithelial cell junctional remodeling and can interact with LIMA1 and Vinculin to maintain the *zonula adherens* (23). Chen et al. (24) identified the substrate of human cell division cycle 14A (CDC14A), the actin regulator LIMA1, by phosphorylated proteomics and the biotin identification proximity assay. LIMA1 phosphorylation regulated by ERK and CDC14A had shown reduced enrichment of α/β-catenin at the cell-cell junctions and downregulation of E-cadherin. Actin kinetic activity was reduced by local regulation of actin rearrangement.

In addition, the actin-stabilizing protein leucine zipper protein 1 (LUZP1) is localized to actin and microtubule structures (25). LIMA1 regulates actin and actin-related protein (myosin Va and ARP2/3) levels by interacting with LUZP1. It also regulates cell migration and ciliogenesis, which is associated with cancer development (26).

#### 2.2.2 Regulation of cell adhesion and metastasis

Cells attach to the network of pre-adsorbed proteins in the extracellular matrix (ECM) or to neighboring cells by interacting with specialized molecules on the cell surface (27). This process is referred to as cell adhesion. Regulation of cell-cell interactions through adhesion junctions is largely dependent on the interaction mediated by E-cadherin and the assembly of a large number of adapter proteins with filamentous actin bundles (28).

LIMA1 acts as an adapter for adhesion junctions and localizes to the integrin adhesion sites of cells in a particularly interesting new cysteine-histidine rich protein 1 (PINCH-1) regulation-dependent manner (29). Depletion of LIMA1 induces the proliferation and migration of keratin-forming cells on collagen and fibronectin both *in vivo* and *in vitro*. Tsurumi et al. (30) showed high expression of the actin cross-linking protein LIMA1 in glomerular thylakoid cells. LIMA1 was concentrated in peripheral actin bundles at local adhesions and formed protein complexes with paxillin. Platelet derived growth factor (PDGF) regulated the interaction of LIMA1 with paxillin by inducing the mitogen-activated protein kinase (MEK)-ERK cascade and also induces subcellular localization of LIMA1 from local adhesion to peripheral folds (13, 30). In addition, ubiquitination of Rab40b-Cullin5 regulates LIMA1 localization and promotes cell migration and invasion by altering adhesion patch and cytoskeletal dynamics (31). Zhang et al. (32) showed that epidermal growth factor (EGF) activated phosphorylation, ubiquitination, and degradation of LIMA1 through an ERK1/2-dependent signaling cascade response. Point mutations in serine residues (serine362 and serine604) of LIMA1 have made it resistant to EGF-induced protein degradation.

Subsequent studies confirmed that LIMA1 was a direct transcriptional target of p53 (14, 33). Knockdown of LIMA1 significantly enhanced cancer cell invasion and partially reversed the p53-induced metastasis of cancer cells (33). It can be speculated that LIMA1 may be a novel prognostic predictor and therapeutic target for tumors. P73, a member of the p53 family, was shown to induce cell cycle arrest and apoptosis, negatively regulating cancer progression (34). Steder et al. (35) found that N-terminal truncation of the P73 gene family produced DNp73, which was frequently upregulated in high-grade tumors. DNp73 inhibited the repression of LIMA1 by interfering with p73, leading to the disruption of intercellular adhesion junctions, which further activated insulin like IGF1R-PKB/STAT3 signaling pathway to initiate the invasion and metastasis cascade response.

#### 2.2.3 Dynamic maintenance of the epithelial mesenchymal transition

Epithelial mesenchymal transition (EMT) is the process by which epithelial cells lose apical-basal polarity and intercellular adhesion and then transform to invasive mesenchymal cells (36). During the transition, proteins characteristic of the epithelial phenotype such as E-cadherin, cytokeratins, or occludin are absent, while N-cadherin, vimentin, or fibronectin are upregulated due to the acquisition of mesenchymal cell characteristics (37). Zhang et al. (32) and Zhitnyak et al. (38) revealed that the actin-binding protein LIMA1 maintained the stability of the circumferential actin bundle, and that EGF induced EMT and increased invasive potential through phosphorylated degradation of LIMA1 leading to active remodeling of the actin cytoskeleton and disruption of intercellular adhesion. It is well known that the regulation of EMT requires the involvement of multiple signaling pathways, including the activation of Wnt/β-catenin, TGF-β, and Notch, among other pathways (39), leading to altered cell morphology, increase in cell motility, and enhanced secretion of growth factors or proteins (40). Subsequently, it was reported that DNp73 exhibited an EMT-like phenotype through dependent downregulation of LIMA1 with loss of E-cadherin and Slug (35). This led to the loss of apical cell polarization and stable cell adhesion, allowing the cells to acquire the ability to invade to promote metastatic potential.

Recent studies support that LIMA1 may regulate the epithelial to mesenchymal transition. Most of these studies suggest that LIMA1 deficiency appears to contribute to the development of EMT and metastasis of cancer cells. LIMA1 is a negative regulator of EMT and invasiveness in prostate cancers, inhibiting E-cadherin, activating β-catenin signaling pathway and enhancing chemoresistance (41, 42). Similarly, LIMA1 depletion was found to promote EMT and induced actin cytoskeleton remodeling in breast cancers (43) and epithelial ovarian cancers (44) significantly enhancing the migration and invasion of epithelial cancer cells both *in vivo and in vitro*. And another study found that the expression of LIMA1 was upregulated in head and neck tumors, driving invasion and metastasis by activating tumor-associated pathways such as PI3K-AKT and JAK-STAT signaling pathways to promote the EMT process (45). It is considered likely that DNA demethylation of the LIMA1 promoter region results in different expression levels of LIMA1 in HNSC.

For future studies on LIMA1 regulation of EMT, we propose constructive and rational research themes. It is possible to quantitatively assess the role of LIMA1 in EMT, explore the molecular mechanisms of LIMA1 regulation of EMT, establish LIMA1 targeting factors and explore their interactions with LIMA1. These studies can predict the cancer biomarkers and patient prognosis associated with LIMA1 and EMT, and help identify novel drugs and therapeutic approaches for cancers.

#### 2.2.4 Modification of the epithelial defense against cancer

During the initial stages of oncogenic mutations, individual cells within the epithelium may undergo transformation (46). Normal epithelial cells can identify the presence of transformed cells and eliminate newly emerging transformed cells by competition, which is known as epithelial defense against cancer (EDAC) (47). Recent studies have shown that transformed cells of RasV12 are extruded from the tip by neighboring epithelial cells (48). This process is the most basic epithelial defense against cancer and involves various non-cellular autonomous changes in normal and transformed cells; however, this is unrelated to the anti-tumor properties of the immune system. The molecular mechanisms behind this phenomenon remain largely elusive. Ohoka et al. (21) found that caveolin-1 (Cav-1) and LIMA1 accumulated in the apical and outer membrane structural domains and in the cytoplasmic matrix of RasV12 transformed cells. LIMA1 acted mainly upstream of Cav-1 to regulate the non-cellular, autonomous activation of myosin II and PKA in RasV12 transformed cells (21, 48). This resulted in extrusion of RasV12 transformed cells from the apical part of the normal epithelial cell monolayer.

In addition, Saitoh et al. (49) showed that Rab5-mediated endocytosis was enhanced in RasV12-transformed cells disrupting cell-to-cell adhesion based on E-cadherin through the dependent regulation of LIMA1. This process affected the accumulation of filamentous proteins in neighboring normal cells, which acted as mechano-sensors exerting the physical forces required for apical extrusion. Importantly, LIMA1 co-accumulated with plectins, microtubules and intermediate filaments in RasV12 transformed cells and positively regulated the apical elimination of transformed cells from the epithelium in a synergistic manner (50). Kasai et al. (51) showed that paxillin induced the acetylation of microtubulin by linking the plectin-LIMA1 complex. It inhibited histone deacetylase 6 (HDAC6) activity, partially rescuing the inhibitory effect of paxillin knockdown on apical extrusion in RasV12 cells (50, 51). In future studies, there is still a need to reveal the mechanism of cytoskeletal organization mechanism of apical extrusion in transformed cells to provide new avenues for establishing novel cancer prevention and treatment.

#### 2.2.5 Promotion of the endothelial cell formation and angiogenesis

Angiogenesis is the process by which endothelial cells form new blood vessels from pre-existing vessels (52). This process includes many complex steps, including the activation, proliferation and migration of endothelial cells, the vascular rings formed by endothelial cell tubes, and the generation of neovascularization and basement membranes (53). Importantly, adherent junctions are required for the remodeling of cellular junctions and the maintenance of vascular endothelial integrity.

The vascular endothelial cadherin (VE-cadherin) is an endothelial cell-specific expressed protein, also known as cadherin 5. VE-cadherin mediates adhesive junctions between adjacent vascular endothelial cells by connecting chain proteins, including α-catenin, β-catenin and γ-catenin, to the actin cytoskeleton (54). Chervin-Pétinot et al. (55) previously presented evidence that LIMA1 co-localized with α-catenin of endothelial cells in the actin cortical loop. LIMA1 attached the VE-cadherin and catenin complexes to the actin cytoskeleton by interacting with α-catenin and actin filaments. It also strengthened cell-cell cohesion by recruiting vinculin, while enhancing endothelial adhesion junctions (55). In addition, ARP2/3 complex dynamically competes with VE-cadherin and a/β-catenin to bind actin filaments (56). This has been shown to be critical in the formation and maintenance of endothelial cell adhesion and integrity mediated by cadherin/catenin complexes.

Subsequently, Hofer et al. (57) used the fluorescent live cell imaging system to explore endothelial cell junctional dynamics under the static and shear stress conditions. By using fluorescent tags (mCherry and EGFP) with self-labelling tags (Halo and SNAP), it was demonstrated that VE-cadherin tagged with EGFP maintained cytoarchitectonic integrity during shear stress-induced junctional remodeling (57). And that two isoforms of LIMA1 were shown to be localized at the cell junctions of vascular endothelial cells (54, 57). However, whether these isoforms have different functions at the cell junctions remains to be investigated. Subsequently, Smith et al. (58) preliminarily verified that LIMA1 subtypes were dependent on stimulation both *in vivo* and *in vitro*. The LIMA1-α expression was increased in endothelial cells during the growth phase; this modulates subcellular localization somewhat and controls protrusion dynamics as driven by actin (58). LIMA1-α controlled the plasma membrane protrusion directly by interacting with the Arp2/3 complex and junction-associated intermittent lamellipodia (JAIL), to increase cell migration and cell junction adhesion (5, 6, 56). The LIMA1-β expression was higher in endothelial cells exposed to aortic endothelium and endothelial cells with high shear stress compared to the vena cava endothelium (58). The magnitude of the LIMA1-β expression in endothelial cells correlates with hemodynamics and acts to induce and stabilize stress fibers. In addition, some investigators conducted *in vitro* studies based on tumor vascular endothelial cells and *in vivo* studies based on animal models (59, 60).

Angiogenesis plays a crucial role in almost all stages of cancer growth, aggressiveness and metastasis. Tumor angiogenesis has been reported to be a hallmark of carcinogenesis (61). Sanders et al. (59) found that overexpression of LIMA1-α could regulate endothelial cell migration, stromal adhesion and formation of new vascular-like structures *in vitro*, and retard tumor formation *in vivo*. It is evident that LIMA1-α has anti-angiogenic effects. Notably, there is was more substantial interaction between LIMA1-α and ERK in endothelial angiogenesis. ERK inhibitors could rescue the tubule formation in HECV cells caused by the overexpression of LIMA1-α (7, 59). Liang et al. (60) also showed that miR-93-5p enhanced the migration and angiogenesis of human umbilical vein endothelial cells (HUVEC) through the downregulation of LIMA1 based on *in vitro* and *in vivo* studies. LIMA1 plays a disruptive role in angiogenesis during cancer development to a certain extent, thus providing a new theoretical basis for the molecular regulation mechanism of the tumor angiogenesis process.

#### 2.2.6 Modulation of the inflammatory response

Endothelial cells are considered key regulators of the inflammatory response. When exposed to the proinflammatory cytokines IL-1β, TNF-α, and IFN-γ, it could lead to local breaks at endothelial cell-cell junctions and exacerbated endothelial barrier dysfunction during the inflammatory response (62). Maucher et al. (63) demonstrated significant changes in the miRNA expression profile of endothelial cells in response to the stimulation by inflammatory cytokines, which may be involved in endothelial permeability regulation. Among them, miR-29a-3p, miR-29b-3p and miR-155-5p expression was significantly increased and suppressed the adhesion protein expression in endothelial cells after transcription. Importantly, the target genes of these miRNAs included β-catenin, p120-catenin and LIMA1, which are key mediators of endothelial cell adhesion junctions (14, 63). These results provide new insights into the dysfunction of the inflammation-induced endothelial barrier and the mechanism of cancer progression.

#### 2.2.7 Maintaining the stability of cell division

Cell division is a fundamental process required for cell proliferation and DNA replication in the majority of organisms, which ensures relative genetic stability (64). During cytokinesis, actin-myosin II contractile loops and septal filaments act synergistically to generate the oval groove and control the contraction of microfilaments constricting it (65). The oval groove is gradually deepened to drive cell membrane invasion causing the parental cells to divide into two daughter cells to complete cytoplasmic division.

Chircop et al. (66) discovered that the actin-binding protein LIMA1 was localized in the oval groove during cytoplasmic division. LIMA1 recruited myosin II, septin 2, small GTP ases, and RhoA to locally accumulate in the oval groove to maintain its formation and contractile ring activity (67). The membrane protein supervillin co-localized with endogenous myosin II and LIMA1 in the oval sulcus and synergistically exerted the functions in regulating actin and microtubule motility (14, 67). Notably, deletion of supervillin could lead to an increase in the number of binucleated and multinucleated cells. Subsequently, Sundvold et al. (68) found that LIMA1 maintained proper cell division by recruiting ACAT-related protein required for viability 1 (Arv1) to the oval groove and driving efficient contraction of the actomyosin ring during the mitotic telomere phase. Both LIMA1 and Arv1 are required for efficient accumulation of myosin at the oval groove. Given that LIMA1 is frequently lost in multiple cancers (13), deletion of LIMA1 affects the recruitment of key cytoplasmic splitting proteins at the oval groove, which leads to the formation of multinucleated cells and increases genomic instability and oncogenicity.

#### 2.2.8 Control of the membrane dynamics

Membrane dynamics is an important component of cellular metabolic processes (69). It includes contraction of the oval groove during cell division, tubularization of plasma membrane receptors, enhancement of laminar lipid formation, cell migration and invasion, and endocytosis during membrane protein sorting and transport (70). These processes are dependent on the structural and functional interconnection of the cell membrane with the cytoskeleton.

As the actin-binding protein, LIMA1 is a key effector molecule mediating pluripotency control of membrane dynamics and cellular metabolism. Duethorn et al. (71) revealed that LIMA1 was ectopically expressed in mouse and human pluripotent stem cells. The LIMA1 expression was transcriptionally controlled by naive pluripotency circuits and could inhibit the membrane vesicle formation (71). As that LIMA1 is required for normal mitochondrial energy in embryonic stem cells and is essential for solid tumor growth.

#### 2.2.9 Regulation of lipid metabolism

Lipid metabolism is an important and complex biochemical reaction in the metabolic processes of human body (72). When the synthesis, decomposition, digestion, absorption, and transport of lipid substances in the body are abnormal, it will cause either too much or too little lipid to be present in each tissue resulting in abnormal lipid metabolism (73). Particularly, cholesterol is an important lipid in the process of lipid metabolism, which synthesizes cholesteryl esters under specific conditions and attaches to the walls of blood vessels and in the liver. High levels of low-density lipoprotein cholesterol (LDL-C) are strongly associated with an increased risk of myocardial infarction and death from vascular diseases due to increased blood cholesterol (74).

Cholesterol variability is controlled by genetic variability. Zhang et al. (75) verified that LIMA1-K306fs SNV was responsible for the low LDL-C variant by Sanger sequencing. The LIMA1 gene mutation was found to be a rare shift mutation in Chinese families with genetically low LDL-C Kazakhs (75). This was the first time that LIMA1 variability was proposed as a newly identified genetic player in the mechanisms controlling cholesterol homeostasis (76). The mutation resulted in low serum LDL-C concentrations and reduced cholesterol absorption in the intestine. Subsequently, Lim et al. (77) similarly demonstrated that cholesterol absorption is reduced with intestinal-specific deficiency of LIMA1 and that inhibition of LIMA1 could reduce LDL-C cholesterol levels. LIMA1 and niemann-pick c1-like 1 (NPC1L1) interactions are required during cholesterol absorption (77). Based on mouse models and tests on human samples, researches had shown that LIMA1 regulated cholesterol transport by recruiting myosin Vb to NPC1L1 to promote efficient intestinal absorption of cholesterol (75, 77). In addition, Su et al. (78) identified genes associated with low disease prevalence based on phenomic, genomic, and metabolomic features, including APOA5, LPL, HIF1A, LIMA1, and others. The association between these genes and lipid metabolism and inflammation is particularly striking, but the exact mechanisms remain unclear.

In the future, it is necessary to study in depth the detailed mechanisms by which LIMA1 controls cholesterol variability to add the meaningful mechanistic insights into the biology of cholesterol absorption. This can provide new therapeutic targets for hypercholesterolemia and abnormal lipid metabolism.

## 3 Roles of *LIMA1* in cancer development and progression

The roles and regulatory mechanisms of LIMA1 gene have received a great deal of scientific attention in a variety of malignancies. This is consistent with the findings that LIMA1 contributes to the maintenance of the epithelial cytoskeleton, supports cell-cell junctions, and regulates lipid metabolism and angiogenesis (6, 14) (**Table 1**). LIMA1 deficiency promotes the disassembly of adhesion-catenin complexes and redistribution of cadherin-catenin complex components (11, 12): it induces remodeling of the actin cytoskeleton and activation of β-catenin signaling, which promotes epithelial to mesenchymal morphology and enhances plasticity and migration of cancer cells (31, 32). Overexpression of LIMA1 has been shown to be effective in manipulating tumor characteristics such as reducing cell growth and cell motility and rendering cells less aggressive (41, 42).

**Table 1:** The molecular and biological functions associated with *LIMA1*.

Processes	The molecules that interacts with LIMA1	Biological significance	References
Actin dynamics	Arp2/3, ERK/MAPK, G-actin, MAL/MRTF, CDC14A, LUZP1, E-cadherin, α-catenin, β-catenin, F-actin, Vinculin, myosin Va	Stabilizing actin filament networks to control actin dynamics	(7, 12, 20–24, 26)
Cell adhesion and metastasis	PINCH-1, PDGF, paxillin, Rab40b-Cullin5, EGF, EPK signaling pathway, p53, DNp73, IGF1R-AKT/STAT3 signaling pathway	Regulation of adherens junctions and initiation of the invasion-metastasis cascade	(29–35)
EMT	EGF, DNp73, E-cadherin, β-catenin, Arp2/3, Slug, PI3K-AKT and JAK-STAT signaling pathway	Bidirectional regulation of the epithelial-to-mesenchymal transition	(32, 35, 38–45)
Epithelial defense against cancer	Cav-1, Myosin II, PKA, Rab5, paxillin	Promotion of apical extrusion in transformed cells	(21, 49–51)
Endothelial dynamics	VE-cadherin, α-catenin, β-catenin, ARP2/3, cLP, JAIL	Mediating the formation and maintenance of endothelial cell adherens junctions and integrity	(54–58)
Angiogenesis	ERK, miR-93-5p	Promotion of new angiogenesis during tumor development	(59, 60)
Inflammatory reaction	MiR-29a-3p, miR-29b-3p, miR-155-5p	Stimulation of inflammatory cytokines, leading to endothelial barrier dysfunction	(63)
Cell division	Arv1, supervillin, Myosin II, septin 2, small GTPases, RhoA	Cooperating to modulate actin and microtubule motor functions, drive the contraction of the cleavage furrow and maintain proper cell division	(66–68)
Membrane dynamics	Actin, β-catenin, p120, the naïve pluripotency transcription factors	Pluripotency control of membrane dynamics and cellular metabolism	(71)
Lipid metabolism	NPC1L1	Regulation of cholesterol transport and promotion of effective cholesterol absorption by the intestines	(75–77)

**Figure 3:**
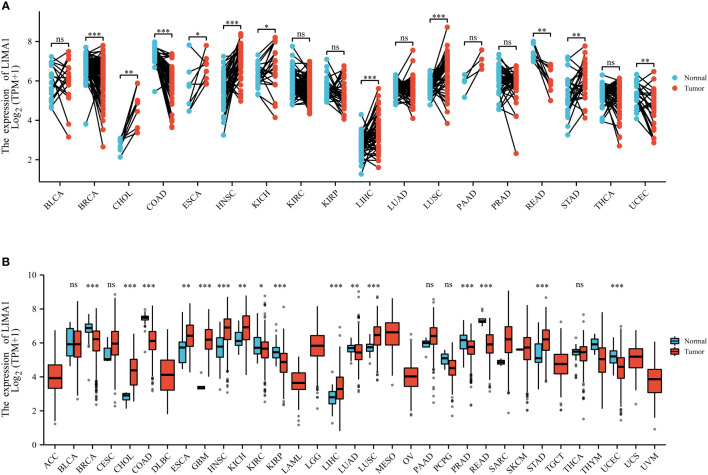
*LIMA1* expression patterns in pan-cancer. **(A)** *LIMA1* expression levels in 33 different types of cancer and non-cancer samples based on *The Cancer Genome Atlas* Database (TCGA) (https://portal.gdc.cancer.gov/). **(B)** LIMA1 expression levels in cancer tissues and its paired normal tissues based on *TCGA* dataset. *p < 0.05, **p < 0.01, ***p < 0.001. "ns" means that there is no statistical difference between the two comparison groups.

**Figure 4:**
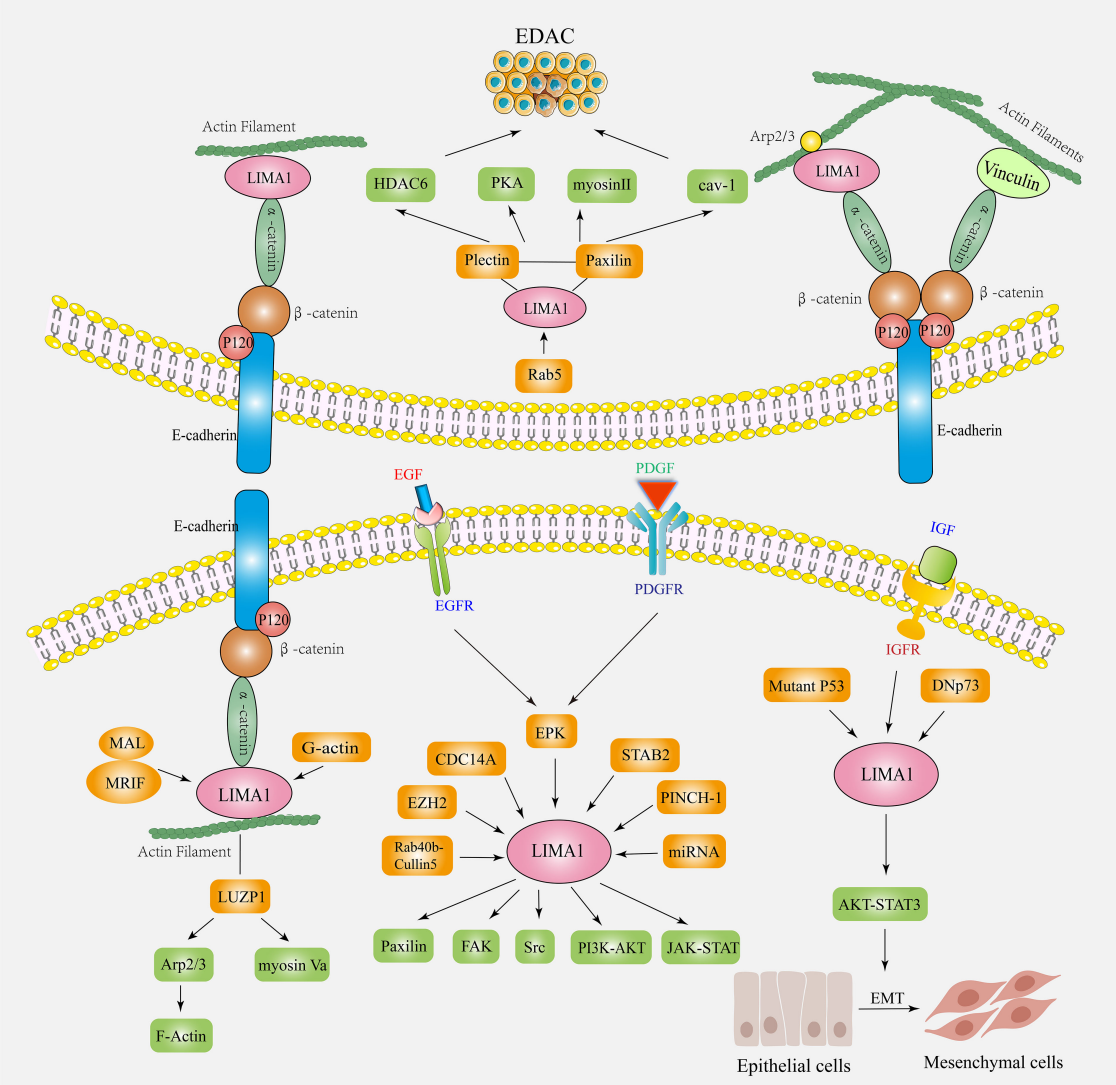
The biological functions and mechanisms of action of LIMA1 in cancers. LIMA1 couples with α-catenin protein, mediates the interaction of cadherin-catenin protein complex with actin. It controls actin dynamics and cell-cell junctions by stabilizing the actin filament networks. LIMA1 acts as a transcriptional target for a variety of factors (ERK, EZH2, CDC14A, Rab40b-Cullin5, STAB2, PINCH-1, miRNA, etc.) by regulating downstream signaling factors (paxillin, plectin, FAK, Src, Cav-1, Myosin II, PKA, etc.) to affect the plasticity and migration ability of cancer cells. LIMA1 deficiency induces the remodeling of the regulatory actin cytoskeleton and activation of signaling pathways, promoting EMT and EDAC, and affects tumor invasion and metastasis.

Recent studies have shown that the LIMA1 gene exhibits frequent downregulation and loss in cancers (**Figure 3**), and it can be involved in the development of malignancy through various mechanisms (13) (**Figure 4**). LIMA1 is associated with the progression and metastasis of various solid tumors including oral cancer (8), esophageal cancer (79), gastric cancer (80), prostate cancer (11, 41, 42, 81), breast cancer (43), ovarian cancer (44), and head and neck squamous cell carcinoma (45, 46), as well as osteosarcoma (82) and lymphoma (83, 84) (**Table 2**). Moreover, LIMA1 particularly plays a crucial role in biological processes such as tumor proliferation, apoptosis, migration, invasion, drug resistance, immune response (79, 80). This suggests that LIMA1 is expected to be a potential prognostic biological marker and therapeutic target for tumor therapy. Therefore, there is an urgent need to further explore the molecular mechanisms by which LIMA1 regulates tumor progression.

**Table 2:** Summary of *LIMA1* expression levels and profiles in cancers.

Cancer types	Cell lines	LIMA1 expression levels	Molecules associated with cancers	Functional roles	References
Esophageal cancer	KYSE150	Downregulated	/	Proliferation and invasion	(79)
Gastric cancer	/	Downregulated	/	Prognosis and NAC chemosensitivity	(80)
Prostate cancer	PC-3	Downregulated	/	Cell invasion, adhesion, growth *in vitro* and tumor development *in vivo*	(11)
	LNCaP, C4-2, MCF-7, ARCaP, PC3	Downregulated	E-cadherin, β-catenin	EMT, migration and invasion	(41)
	PC-3, LNCaP, CA-HPV-10	Downregulated	Paxillin, FAK/SRC signaling pathway	Proliferation, migration and invasion	(42)
	C4-2, C4-2B, ARCaP	Downregulated	EZH2	Metastasis and chemoresistance	(81)
Breast cancer	MDA MB-463, MDA MB-435s, MDA MB-436, MCF10A, MCF-7, ZR 7-51, MDA MB-468, BT-482, BT474, BT549, MDA MB-157 ANDMDA, MB 231, IBTG3 MRC5	Downregulated	ERK	Proliferation, migration and invasion *in vitro* and tumor development *in vivo*	(43)
Epithelial ovarian cancer	SKOV3, COV504	Downregulated	/	Cell growth, migration, invasion and adhesion	(44)
Head and neck squamous cell carcinoma	CAL27, HSC4	Upregulated	PI3K/AKT and JAK-STAT signaling pathway	Tumor metastasis, hypoxia, angiogenesis, and EMT	(45)
Osteosarcoma	KHOS, MNNG, U2OS	Downregulated	SATB2, RhoA, Rac1, FAK and Paxillin	Adhesion, migration and invasion	(82)
Mucosa-associated lymphoid tissue lymphoma	293T, 293FT, BJAB-Tet-On, SSK41	Downregulated	API2-MALT1, LIM domain-only fragment	Proliferation, migration, invasion, antibiotic resistance and B-cell oncogenesis	(83)
Diffuse large B-cell lymphoma	BJAB, SUDHL4, HEK 293T	Downregulated	MiR-142	Cell growth	(84)

"/" means that this aspect has not been studied out or or not covered in the relevant reference.

## 4 Expression of *LIMA1* and its regulatory mechanisms in digestive system tumors

Digestive system cancers are one of the most common leading causes of cancer deaths worldwide, with high morbidity and mortality (85). It is difficult to detect cancers at an early stage due to lack of effective biomarkers (86). Therefore, identification of potentially effective biomarkers can help in the early diagnosis of such cancers. The current research hot-spots on LIMA1 in digestive system tumors are mainly focused on esophageal cancer (79) and gastric cancer (80). While our laboratory has validated the regulatory mechanism of LIMA1 with liver cancer. LIMA1 is expected to be a new biomarker for tumor diagnosis, prognostic biomarker and targeted therapy to improve survival of cancer patients in the future.

### 4.1 *LIMA1* and esophageal cancer

Esophageal cancer is the most aggressive of all malignancies of the gastrointestinal tract. Esophageal cancer is prone to recurrence and metastasis, and the current five-year survival rate for patients remains low due to its high morbidity and mortality (87). Liu et al. (79) used the quantitative polymerase chain reaction (q-PCR) method to determine the aberrant expression of LIMA1-α transcripts in human esophageal tissues (tumor, paraneoplastic, and normal). It was also shown that overexpression of LIMA1-α reduced the aggressiveness of the esophageal cancer cell line *KYSE150* and attenuated the rate of cell invasion and growth *in vitro*. This is consistent with previous studies showing that LIMA1-α expression is frequently downregulated or lost in a variety of different cancer cell lines, including oral cancer cells (8), prostate cancer cells (11, 41), and breast cancer cell lines (43). There was a negative correlation between LIMA1-α expression levels and tumor grade, lymph node status, tumor stage, and whether patients remained disease-free or died from cancers (79). Combined with the findings of LIMA1-α inhibition of cell growth and invasion, it was found that LIMA1-α downregulation has a predictive value. This highlights the potential of LIMA1 as a prognostic indicator and that this molecule may act as a protective factor.

### 4.2 *LIMA1* and gastric cancer

Gastric cancer is one of the most common and highly aggressive malignancies worldwide. It accounts for more than 1 million new cases each year and remains the third leading cause of cancer death (88). Surgical resection is the best option for early-stage gastric cancer, while chemotherapy is mainly used in the intermediate and late stages of the disease (89), however, there are many reports of treatment failure in gastric cancer due to chemotherapy resistance. Combined with the low rate of early diagnosis, limited treatment and tumor heterogeneity, the prognosis of gastric cancer remains poor.

Gong et al. (80) explored the correlation between LIMA1 transcript expression and patient clinicopathological factors and its importance in neoadjuvant chemotherapy (NAC) responsiveness through two gastric cancer cohorts collected from Beijing Cancer Hospital. Additional studies have shown that the effect of NAC plays a key role in the prognosis of gastric cancer patients (90). Initially larger gastric cancer cohort to assess the association between LIMA1 expression and clinico-pathological features and prognosis. A second smaller cohort containing patients receiving NAC was evaluated to explore the role of LIMA1 in response to chemotherapy (14, 80). LIMA1 negatively regulates biological function in gastric cancers requiring NAC and may promote longer overall survival. Moreover, LIMA1 inhibits deep tumor infiltration and promotes differentiation, playing an important role in chemotherapy responsiveness. It is suggested that LIMA1 may be a potential prognostic indicator for gastric cancer.

Therefore, further studies and larger cohorts are necessary in the future the better to understand the role of LIMA1 in gastric cancer, particularly regarding its involvement in chemoresistance and therapeutic response. This will provide new targets for biomarkers or therapeutic strategies related to LIMA1 in gastric cancer.

### 4.3 *LIMA1* and hepatocellular carcinoma

Hepatocellular carcinoma (HCC) is a major global problem, with viral hepatitis B and C infection, alcohol abuse and metabolic disorders being multiple risk factors for the development of cirrhosis and HCC (91). Due to the lack of symptoms in the early stages of HCC, most patients are diagnosed with metastasis in the late stages of the disease (92). Therefore, the need to find promising biomarkers for HCC diagnosis is urgent.

Existing studies suggest that LIMA1 plays an inhibitory role in most malignant tumors, but some studies has been reported that LIMA1 exerts an oncogenic effect in the head and neck tumors (41–43, 45, 81). However, the role and regulatory mechanisms of LIMA1 in hepatocellular carcinoma are still not well understood, and its biological function and clinical value need to be further explored. Increasing research evidences imply that LIMA1 affects cancer progression by regulating important biological processes such as actin dynamics, cell adhesion and metastasis, angiogenesis and lipid metabolism (5, 13, 14). There are no published articles on the effect of LIMA1 on the malignant phenotype of hepatocellular carcinoma. Our study found that the LIMA1 was highly expressed in hepatocellular carcinoma tissues and cell lines. Overexpression of LIMA1 enhanced the migration and invasion of hepatocellular carcinoma cells, which conflicted with our previous knowledge of LIMA1. Subsequently, we will focus on the specific mechanisms of how LIMA1 regulates HCC, which may provide novel therapeutic targets for the treatment of HCC patients.

## 5 Expression of *LIMA1* and its regulatory mechanisms in urinary system tumors

Research on LIMA1 in urologic system tumors is currently focused on prostate cancer. Prostate cancer (PCa) is the most common type of solid tumor in men and the second most common cause of cancer-related deaths in men worldwide (93). Survival of PCa patients depends on early disease diagnosis and effective treatment options, however, lack of specificity to serum prostate specific antigen has been shown to lead to overdiagnosis and overtreatment of PCa (94). A growing number of studies have shown consistent results that LIMA1 is a negative regulator of EMT and aggressiveness, and LIMA1 is negatively correlated with PCa progression (11, 41, 42, 81).

Sanders et al. (41) cloned the full-length human LIMA1 cDNA gene into an expression vector and transfected the human prostate cancer cell line PC-3. Overexpression of LIMA1 in PCa resulted in reduced growth potential both in vitro and in vivo, and decreased cell invasiveness and extracellular matrix adhesion in multiple model assays. In addition, Collins et al. (42) generated LIMA1-α overexpression models and confirmed that overexpression of LIMA1-α reduced cell growth, migration and invasion and affected transcription and protein expression of paxillin, focal adhesion kinase (FAK) and tyrosine protein kinase (Src). Several earlier studies found that LIMA1 was associated with cell-cell adhesion through the interaction of cadherin-catenin complexes bound to F-actin (6, 13). Studies have also suggested a possible link between this molecule and Paxillin and a possible role in regulating cell adhesion to the extracellular matrix (51). Thus, it is evident that these studies confirmed the importance of LIMA1 in prostate cancer and further highlighted the importance of LIMA1 in regulating the growth and aggressiveness of prostate cancer cells.

Subsequently, Zhang et al. (11) identified a significant downregulation of LIMA1 after EMT by quantitative proteomics using an experimental model of prostate cancer metastasis. Biochemical and functional analyses showed that LIMA1 was a negative regulator of EMT and PCa cell invasiveness (13). LIMA1 downregulation significantly could disrupt epithelial architecture, induce actin cytoskeleton remodeling, affect specific gene expression profiles, and activate the pro-EMT program (95). In recent years, Wu et al. (81) established androgen-repressed prostate cancer (ARCaP) cells with temporary or permanent knockdown of LIMA1 to determine the function of LIMA1 in PCa EMT and aggressiveness. The use of the ARCaP EMT model was demonstrated by the understanding of LIMA1 biology and the ARCaP model in the discovery of new drugs for the prevention and treatment of prostate cancer metastasis.

Future studies will need to focus on the generation and efficacy of recombinant forms of LIMA1 for the treatment of prostate cancer both in vitro and in vivo, thus facilitating the identification of new “druggable” therapeutic targets for the treatment of metastatic prostate cancer to help design new therapeutic strategies.

## 6 The expression of *LIMA1* and its regulatory mechanisms in breast and reproductive system tumors

### 6.1 *LIMA1* and breast cancer

Breast cancer is the most common malignancy that threatens the health of women worldwide. The incidence of breast cancer is increasing year-on-year, but the prognosis for patients remains pessimistic (96). Jiang et al. (43) first identified downregulation of the LIMA1-α expression in breast cancer cells and tissues. The LIMA1-α expression was associated with reduced growth of breast cancer cells both in vitro and in vivo and inhibited cell migration and invasiveness by relying on the ERK1/2 signaling pathway. Previous studies have shown that LIMA1 acts as a link between the E-Cadherin-β-Catenin -α-Catenin complex and actin filaments to maintain epithelial phenotype, stabilize the actin cytoskeletal network, and maintain functional epithelial junctions (23, 31). EGF phosphorylates LIMA1 thereby leading to LIMA1 degradation. Furthermore, there is a clear clinical correlation between the LIMA1 expression and tumor grade, lymph node status and tumor stage of breast cancers (43). It is evident that LIMA1 is a negative regulator of breast cancer cell migration. Upregulation of LIMA1 leads to the suppression of cell invasion ability, migration ability and growth rate in vitro and in vivo. Thus, it is hypothesized that LIMA1 is an important prognostic indicator and could be considered as an important target during targeted breast cancer therapy in the future.

### 6.2 *LIMA1* and epithelial ovarian cancer

Epithelial ovarian cancer (EOC) is one of the most lethal gynecological malignancies. It is diagnosed at the late stage in most women which explains the poor prognosis of this malignancy (97). Liu et al. (44) used reverse transcription-polymerase chain reaction (RT-PCR) and immune-histochemistry (IHC) methods to validate the downregulation of the LIMA1-α expression in human ovarian cancer tissues and cell lines at the mRNA and protein levels. Compared with control cells, the knockdown of LIMA1-α caused a significant increase in ovarian cancer cell growth, adhesion, invasion and migration ability. The inhibitory effect of LIMA1 on ovarian cancer cell growth is in line with findings in breast cancer (43), prostate cancer (41, 42), esophageal cancer (79), and endothelial cell lines (54, 55). This was the first article to confirm the LIMA1-α expression in human epithelial ovarian cancer tissues and its effect on the biological behavior of epithelial ovarian cancer cell lines (44).The precise molecular mechanism by which LIMA1-α inhibits the epithelial ovarian cancer phenotype remains unknown. However, relevant studies have provided conclusive evidence that reduction of LIMA1 has the potential to regulate cancer cell migration and invasion by disrupting cell-cell adhesion of adherens junctions, reducing E-cadherin expression and enhancing the EMT-like phenotype (31, 35). These findings suggest that prevention of LIMA1 degradation or partial restoration of the LIMA1 expression may be a novel strategy for the treatment of invasive ovarian cancer growth and metastasis. LIMA1 has the potential to be used as a prognostic predictor and this molecule, in patients with epithelial ovarian cancer, acts as a protective factor.

## 7 Expression of *LIMA1* and its regulatory mechanisms in the head and neck tumors

Oral cancer is the most common tumor of the head and neck region and is the sixth most common tumor worldwide (98). The incidence of oral cancer has increased significantly due to the oncogenic effect of human papillomavirus (HPV). It has one of the highest mortality rates and this has remained the case for more than 20 years (99). Given its difficulty in early diagnosis, susceptibility to metastasis, and poor prognosis, many studies aimed to find more sensitive, specific, and valuable tumor markers. LIMA1 was initially found to be a downregulated gene in oral cancer (8). Subsequently, Wirsing et al. (100) studied prognostic markers (CALML5, CD59, and LIMA1) selected from pathological profiles in patients with head and neck cancers. They also performed an unbiased analysis of these prognostic markers at the mRNA and protein levels in 121 oral cancer patients. Only CALML5 showed significant prognostic value, while the prognostic value of CD59 and LIMA1 could not be validated in this cohort, emphasizing the necessity to assess the head and neck cancer specificity by subgroup analysis (100).

Notably, another study has identified LIMA1 overexpression in the head and neck squamous cell carcinoma (HNSC) (45). High expression of LIMA1 is associated with carcinogenesis and predicts a poor prognosis, especially in HPV-negative and TP53-mutated HNSC. HPV is involved in 25% of HNSC cases and is strongly associated with prolonged survival in HPV-positive patients (45). The increased levels of TP53 mutation may lead to dysfunction of this gene, which has been shown to have oncogenic effects in several cancer types (101). It has been reported that TP53-induced LIMA1 inhibits cell invasion and that TP53 mutations lead to upregulation of LIMA1 expression levels, leading to EMT and further driving tumor invasion and metastasis (33, 45). DNA demethylation of the LIMA1 promoter region may affect its expression upregulation, positively correlating with tumor metastasis, angiogenesis, and EMT. These could illustrate the heterogeneity of LIMA1 expression and the diversity of its functions, and more detailed studies of specific regulatory mechanisms are needed in the future to provide new insights for the development of new biomarkers and personalized cancer therapy.

## 8 Expression of *LIMA1* and its regulatory mechanisms in other tumors

### 8.1 *LIMA1* and osteosarcoma

Osteosarcoma is the most common primary malignancy among bone tumors, with peak incidence concentrated in children and adolescents (102). Osteosarcoma has a high propensity for local invasion and metastasis (103). Seong et al. (82) identified genes differentially regulated by special AT-rich-binding protein 2 (SATB2), including LIMA1, by microarray analysis. Silencing LIMA1 expression resulted in reduced cell adhesion and increased stress fibers in knockdown SATB2 cells compared with control cells, partially rescuing the reduced invasive phenotype of knockdown SATB2. It is evident that SATB2-mediated invasion may be due to interference with LIMA1 expression, which is a key mediator of SATB2-regulated osteosarcoma invasion. In addition, the novel finding indicated that LIMA1 regulated paxillin levels and phosphorylation (82) and phosphorylated paxillin was known to affect variations of adherent patches (30, 51). These suggest that LIMA1 may regulate osteosarcoma invasion by regulating cell adhesion and adherent patch changes.

In future work, there is a need to explore in depth the transcriptional regulatory mechanisms of SATB2 regulatory genes and signaling pathways, including LIMA1 that control the effect of the actin cytoskeleton on motility and invasion. This may help to discover targeting proteins for metastatic osteosarcoma and other cancers with high expression of SATB2.

### 8.2 *LIMA1* and mucosa-associated lymphoid tissue lymphomas

Mucosa-associated Lymphoid Tissue (MALT) lymphoma is a relatively inert non-hodgkin lymphoma (NHL) containing B cells (104). Its pathogenesis progresses in association with chronic inflammation (105). A gene fusion between apoptosisinhibitor-2 (API2) on chromosome 11 and the mucosa-associated lymphoid tissue translocation gene 1 (MALT1) on chromosome 18 formed the API2-MALT1 oncogenic chimera (106). This is the most common chromosomal translocation in MALT lymphomas and has disrupted the function of the MALT1 gene. Using an efficient strategy related to tandem mass spectrometry, Nie et al. (83) identified LIMA1 as a novel interactor and substrate for API2-MALT1 chimeric cystathionase. Interestingly, it was also shown that API2-MALT1-mediated protein hydrolysis produced a LIM domain-only containing oncogenic characteristic fragments both in vivo and in vitro (83), implying that API2-MALT1 converted LIMA1 into an oncogenic LIM domain-only-like protein in MALT lymphoma.

The oncogenicity of B cells was observed through LIMA1-α cleavage and RNA deletion that can be mediated by API2-MALT1 (107). This suggests that LIMA1-α functions as a putative tumor suppressor in B cells akin to its inhibitory function in epithelial cells. Overall, specific inhibition of the interaction between API2 and LIMA1 may facilitate the development of targeted therapies against API2-MALT1 positive lymphomas.

### 8.3 *LIMA1* and diffuse large B-cell lymphoma

Diffuse large B-cell lymphoma (DLBCL) is the most common subtype of aggressive lymphoma. DLBCL is frequently found in most cases of NHL that are aggressive or moderate to highly malignant (108). The pathology of DLBCL is characterized by a diffuse growth of large lymphocytes and a characteristic distribution of tumor cells in the perivascular space (109).

Based on miRNA analysis of DLBCL, Kwanhian et al. (110) identified microRNA-142 (miR-142) as the human microRNA gene that was consistently mutated in 20% of cases with primary DLBCL. Deletion of miR-142 in knockout mice has been reported to result in dysregulated lymphangiogenesis and immunodeficiency (111). Menegatti et al. (84) validated the effect of miR-142 inactivation on protein expression in DLBCL by CRISPR/Cas9 knockdown of miR-142 in DLBCL cell lines BJAB and SUDHL4. MiR-142 knockdown induced a consistent upregulation of genes or proteins that were oncogenic and a downregulation of genes or proteins associated with immunodeficiency responses required for MHC-I presentation. Among them, CCNB1, LIMA1, and TFRC were identified as potential targets and novel targets for miR-142 knockdown cell lines (84). The role of miRNAs to influence cellular transcriptional regulatory networks was further enhanced.

## 9 Conclusions and future prospects

Notably, using human tumor specimens as the “gold standard”, the EPLIN expression was found to be negatively correlated with clinical lymph node metastasis in a variety of solid tumors (**Table 3**). The role of LIMA1 in malignancy progression has expanded from a tumor suppressor that acts primarily in early disease stages to a metastasis suppressor that may act in late stages to prevent and delay the invasion and spread of primary cancer cells.

**Table 3:** Clinical pathological features and prognostic significance of LIMA1 in cancers.

Cancer types	Samples	LIMA1 expression	Related clinical pathological features	Prognostic significance	Ref.
Esophageal cancer	Tumor tissues, normal tissues adjacent to tumor and normal tissues for each patient (no specific quantity is mentioned)	Downregulated	Histological grade, survival status, nodal status, and TNM stage	A tumor suppressor with prognostic value	(79)
Gastric cancer	The first cohort contained 320 gastric cancer tissues and 175 paired normal tissues, and the second cohort contained 78 gastric cancer tissues and 80 normal tissue sample regarding patient responsiveness to NAC	Downregulated	TNM stage, T stage, nodal involvement, metastasis status, invasion, embolism and chemosensitivity	A prognostic factor associated with NAC chemosensitivity.	(80)
Prostate cancer	Human PCa tissue specimens (no specific quantity is mentioned)	Downregulated	Clinical lymph node metastasis	A negative regulator	(11)
	20 prostate tumor and 11 normal prostate tissue samples	Downregulated	/	A tumor suppressor	(42)
	69 primary PCa cases and 10 cases composed of benign prostatic glands and stroma	Downregulated	Histological grade	A predictor of tumor aggressiveness and poor prognosis	(81)
Breast cancer	120 tumor and 32 normal mammary tissues	Downregulated	Histological grade, TNM stage, nodal involvement, overall and disease-free survival	A potential tumor suppressor and had a prognostic value	(43)
Epithelial ovarian cancer	30 epithelial ovarian serous carcinomas, 15 samples were non-metastatic and 15 had lymph node or omentum metastases.	Downregulated	Metastasis status	A prognostic indicator and a protective factor	(44)
Head and neck squamous cell carcinoma	519 tissues of head and neck squamous cell carcinoma and 44 normal tissues from the TCGA cohort	Upregulated	TNM stage, clinical stage, radiation therapy, overall survival, disease specific survival	An independent prognostic predictor	(45)
Mucosa-associated lymphoid tissue lymphoma	API2–MALT1-positive and -negative MALT lymphoma tissues (no specific quantity is mentioned)	Downregulated	/	Aggressive clinical behavior with poor clinical outcome	(83)

"/" means that this aspect has not been studied out or or not covered in the relevant reference.

With the deepening of the LIMA1 gene research, LIMA1 has been recognized as being actively involved in cancer cell signaling, possibly through multiple protein interactions associated with cancer progression, in addition to its function as a structural protein. Moreover, LIMA1 is also involved in apical elimination, cilia growth, cholesterol uptake, cellular metabolism, angiogenesis, and endothelial cell dynamics. This provides an additional avenue to explore the implications and mechanisms of LIMA1 in a broader context. Expanding our exploration of multiple areas of this important molecule, LIMA1, in the future will increase its therapeutic potential and help design new metastatic disease prevention and treatment.

## Author contributions

XW and CZ drafted and revised the original draft preparation. HS and JY revised the manuscript. XZ and YY collected relevant papers. LZ and JH reviewed the article. XW designed tables and charts. All authors contributed to the article and approved the submitted version.

## Glossary

**Table:**

LIM domain and actin-binding protein 1	LIMA1
epithelial protein lost in neoplasms	EPLIN
sterol regulatory element binding protein 3	SREBP3
Dual Specificity Phosphatase 5	DUSP5
Vinculin	VCL
Catenin Delta 1	CTNND1
Catenin Alpha 1	CTNNA1
Catenin Beta 1	CTNNB1
Signal Induced Proliferation Associated 1 Like 1	SIPA1L1
Cadherin 17	CDH17
Nexilin F-Actin Binding Protein	NEXN
Interferon Related Developmental Regulator 2	IFRD2
actin-related protein complex 2/3	Arp2/3
extracellular signal-regulated kinase	ERK
mitogen-activated protein kinase	MAPK
megakaryoblastic acute leukemia	MAL
myocardin related transcription factor	MRTF
cell division cycle 14A	CDC14A
leucine zipper protein 1	LUZP1
extracellular matrix	ECM
particularly interesting new cysteine-histidine rich protein 1	PINCH-1
platelet derived growth factor	PDGF
mitogen-activated protein kinase	MEK
epidermal growth factor	EGF
insulin like growth factor 1 receptor	IGF1R
Protein Kinase B	PKB
signal transducer and activator of transcription 3	STAT3
Epithelial mesenchymal transition	EMT
epithelial defense against cancer	EDAC
Caveolin-1	Cav-1
histone deacetylase 6	HDAC6
vascular endothelial cadherin	VE-cadherin
junction-associated intermittent lamellipodia	JAIL
human umbilical vein endothelial cells	HUVEC
ACAT-related protein required for viability 1	Arv1
low-density lipoprotein cholesterol	LDL-C
Niemann-Pick C1-like 1	NPC1L1
The Cancer Genome Atlas	TCGA
quantitative polymerase chain reaction	q-PCR
neoadjuvant chemotherapy	NAC
Hepatocellular carcinoma	HCC
focal adhesion kinase	FAK
Androgen-Repressed Prostate Cancer	ARCaP
Epithelial ovarian cancer	EOC
reverse transcription-polymerase chain reaction	RT-PCR
immune-histochemistry	IHC
head and neck squamous cell carcinoma	HNSC
special AT-rich-binding protein 2	SATB2
Mucosa-associated Lymphoid Tissue	MALT
non-hodgkin lymphoma	NHL
apoptosisinhibitor-2	API2
the mucosa-associated lymphoid tissue translocation gene 1	MALT1
Diffuse large B-cell lymphoma	DLBCL
microRNA-142	miR-142
